# Phase Variation Leads to the Misidentification of a *Neisseria Gonorrhoeae* Virulence Gene

**DOI:** 10.1371/journal.pone.0072183

**Published:** 2013-08-16

**Authors:** Mark T. Anderson, H. Steven Seifert

**Affiliations:** Department of Microbiology-Immunology, Northwestern University, Feinberg School of Medicine, Chicago, Illinois, United States of America; University of Illinois at Chicago College of Medicine, United States of America

## Abstract

*Neisseria gonorrhoeae* is the causative agent of gonorrhea and an obligate pathogen of humans. The Opa proteins of these bacteria are known to mediate attachment and internalization by host cells, including neutrophils. The Opa protein repertoire of a typical *N. gonorrhoeae* isolate is encoded on ∼11 genes distributed throughout the chromosome and is subject to stochastic changes in expression through phase variation. Together, these characteristics make Opa proteins a critical yet unpredictable aspect of any experimental investigation into the interaction of *N. gonorrhoeae* with host cells. The goal of this study was to identify novel virulence factors of *N. gonorrhoeae* by assessing the contribution of a set of uncharacterized hydrogen peroxide-induced genes to bacterial survival against neutrophil-mediated killing. To this end, a strain harboring an engineered mutation in the NGO0322 gene was identified that exhibited increased sensitivity to neutrophil-mediated killing, enhanced internalization by neutrophils, and the ability to induce high levels of neutrophil-generated reactive oxygen species. Each of these phenotypes reverted to near wild-type levels following genetic complementation of the NGO0322 mutation. However, after immunoblot analysis of Opa proteins expressed by the isogenic parent, mutant, and genetically complemented strains, it was determined that phase variation had resulted in a disparity between the Opa profiles of these strains. To determine whether Opa phase variation, rather than NGO0322 mutation, was the cause of the observed neutrophil-related phenotypes, NGO0322 function was investigated in *N. gonorrhoeae* strains lacking all Opa proteins or constitutively expressing the OpaD variant. In both cases, mutation of NGO0322 did not alter survival of gonococci in the presence of neutrophils. These results demonstrate the importance of controlling for the frequent and random variation in Opa protein production by *N. gonorrhoeae* when investigating host cell interactions.

## Introduction

*Neisseria gonorrhoeae* is a pathogen that strictly infects humans, causing the disease gonorrhea. During infection, *N. gonorrhoeae* (gonococci) associate with innate immune cells, including neutrophils, following a robust inflammatory response that is the hallmark of symptomatic infection. Despite this host response viable gonococci can be recovered from the inflammatory exudate [1] and thus interaction with the innate immune system can be modeled experimentally by infecting primary neutrophils in the laboratory. A fundamental goal of *N. gonorrhoeae* research has been the investigation of mechanisms by which this organism is able to survive the antibacterial defenses of innate immune cells and maintain a disease state. As with many investigations into bacterial physiology, this study followed the paradigm of identifying genes of interest then determining what effect mutations within these genes have on a particular bacterial phenotype, in this case the ability to survive in the presence of neutrophils. However, this type of investigation requires some special considerations in the case of *N. gonorrhoeae*, owing to the tremendous capacity for stochastic changes in protein expression (phase variation) in this organism. The importance of gonococcal Opa surface adhesins and type IV pili in mediating interactions with host cells has been a long-appreciated characteristic of *N. gonorrhoeae* biology [2]. However, an often overlooked issue when constructing isogenic mutant strains for the study of host interactions is the impact that random sequence variation has on such interactions, as has previously been observed for antigenic variation of type IV pili [3]. The Opa proteins are also variable in that they switch frequently and reversibly between an “on” or “off” sequence arrangement at any of the ∼11 *opa* alleles encoded on a typical gonococcal chromosome [4]–[6].

Phase variation is a well-characterized mechanism by which *N. gonorrhoeae* and other bacterial species stochastically modulate production of proteins. Numerous bacterial species employ phase variation, by a variety of mechanisms, as a means of introducing phenotypic diversity within a population [7]. Slipped-strand mispairing during chromosomal replication is one means by which phase variation can occur and there are many examples of surface proteins expressed by human pathogens that are altered in this manner, including; *Bordetella pertussis* fimbriae [8], *Haemophilus influenzae* lipopolysaccharide [9], [10] and fimbriae [11], and a *Helicobacter pylori* adhesin [12], [13]. Phase variation of the *N. gonorrhoeae* Opa proteins occurs through changes in the number of pentameric repeats (CTCTT) within the leader peptide encoding sequence of each *opa* gene copy, leading to restoration or disruption of the *opa* reading frame [14]. The frequency of phase variation at these loci has been reported to be ∼10^−3^ per cell per generation [15], resulting in a high potential for surface polymorphism in any given population, including short-lived laboratory cultures. In addition to the *opa* genes, *N. gonorrhoeae* have numerous other genes for which evidence of phase variation exists [16], including type III restriction modification genes that further influence the expression of an extensive gene repertoire [17].

Recent work by two groups have characterized the impact of Opa proteins on both bacteria-bacteria interactions [18] and bacteria-neutrophil interactions [19] by constructing and analyzing *N. gonorrhoeae* strains that were genetically engineered to be devoid of Opa proteins. These strains represent valuable laboratory tools that provide a genetic background to investigate the contribution of these and other proteins to *Neisseria* virulence. Given the inherent difficulty in assessing host cell interactions in the presence of Opa proteins it is tempting to eliminate the influence of Opa proteins altogether, but it is worth noting that there is strong selective pressure for Opa expression during human infection [20]–[22]. Ideally then, it would be beneficial to examine the contribution of novel *Neisseria* virulence factors in the context of Opa expression.

Reactive oxygen species (ROS) are produced by neutrophils in response to bacterial infection and represent one type of antimicrobial defense mechanism employed by these cells. It has been previously shown that gonococci elicit ROS production by infected neutrophils, at least in part due to Opa-mediated activation of the CEACAM3 receptor [23], but that the bacteria are resistant to this type of assault [24]–[26]. The means by which *N. gonorrhoeae* resist neutrophil oxidative stress are largely unknown as several candidate bacterial genes with anti-oxidant function have been shown to be dispensable for survival in the presence of neutrophils [26], [27]. It has also been shown that *N. gonorrhoeae* lacking Opa proteins have the capacity to suppress the oxidative burst of neutrophils and it is reasoned that this may be one means by which the bacteria evade the oxidative response [28]. As there is strong selective pressure for the expression of Opa proteins [20]–[22], it seems unlikely that suppression of the oxidative burst is the sole means by which bacteria survive neutrophil interactions. Several of the known bacterial ROS-related genes are transcriptionally activated in the presence of hydrogen peroxide as determined by transcriptional microarray [29]. Interestingly, the same study determined that there were many ROS-inducible genes that have no predicted antioxidant function. Two of these genes, NGO1686 and *recN*, have additionally been demonstrated to be important for bacterial resistance to nonoxidative neutrophil-mediated killing [26], [29]. ROS perception by *N. gonorrhoeae* was therefore postulated to serve as a bacterial survival signal and thus additional novel virulence factors may be discovered by investigating the contribution of uncharacterized ROS-responsive genes to gonococcal survival in the presence of neutrophils. In the course of this analysis a strain containing an engineered mutation in the NGO0322 gene was found to have increased sensitivity to neutrophil-mediated killing. Manipulation of the strain to achieve genetic complementation of the NGO0322 mutation resulted in near complete restoration of the PMN-related phenotypes associated with the original NGO0322 mutation. However, careful analysis of the Opa protein expression profile of the NGO0322 mutant strain compared to control strains indicated that the PMN sensitivity phenotype was the result of Opa phase variation and not the function of the NGO0322 gene product.

## Materials and Methods

### Bacterial Strains and Culture Conditions

Bacterial strains used in this study were derivatives of the FA1090 clinical isolate engineered to contain a mutation in the guanine nucleotide repeat region adjacent to the *pilE* locus (FA1090 NV) [30]. The result of this mutation is that the strain maintains a greatly reduced frequency of pilin antigenic variation. All gonococcal strains were screened for the 1-81-S2 variant of type IV pili [31] in an effort to further reduce the influence of *pilE* sequence variation on the phenotypes tested. The FA1090 NV strain in which all 11 *opa* gene copies were sequentially deleted (Opaless) and the derivative that harbors a non-variable copy of the *opaD* gene (Opaless, *opaD* ^+^_NV_) were kindly provided by A. Criss [19]. *Escherichia coli* strains DH5α (Invitrogen), TOP10 (Invitrogen), and a DH10B derivative (BH10B) harboring a *pcnB* mutation to reduce plasmid copy number [32] were used for routine cloning purposes. *N. gonorrhoeae* strains were maintained on gonococcal medium base (Difco) or in liquid broth modified with Kellogg supplements as described previously [33]. Bacteria used in phenotypic analyses were propagated in liquid culture using a previously described method [34] to obtain a uniform population in mid-exponential phase.

### Construction of Mutant Strains

Hydrogen peroxide-induced genes (Table 1) were amplified from FA1090 genomic DNA by PCR using the KOD polymerase (Novagen) according to the manufacturer’s recommendations. The resulting PCR products were ligated to one of the following plasmids based on copy number and restriction site compatibility; pCR-Blunt (Invitrogen), pSMART LC Amp (Lucigen), or pGEM3Z (Promega). The EZ-Tn*5*<KAN-2> transposon (Epicentre) was used to generate random insertion mutations *in vitro* in plasmids harboring peroxide inducible genes. Kanamycin resistant *E. coli* transformants were screened by PCR and the location of the transposon insertion was determined by sequencing. Plasmids containing transposon insertions in the desired locations were used to transform *N. gonorrhoeae* as described previously [35]. Gonococcal transformants were confirmed to contain the appropriate transposon insertion by PCR and genomic DNA was used to backcross the mutation into the parental FA1090 NV strain prior to further analysis. Each mutant was confirmed to be Opa^+^ by western blot (see below). The sequences of oligonucleotide primers used in the construction of mutant strains are available upon request.

**Table 1:** Relative transcript levels of select *N. gonorrhoeae* genes after exposure to hydrogen peroxide.

ORF^a^	Predicted function	Fold induction^b^	Mutatable^c^
NGO0108	NADPH-dependent FMN reductase	7.0±2.2	no
NGO0114	glutaredoxin	6.7±2.5	yes
NGO0322	FMN-binding protein	24.8±12.8	yes
NGO0376	peptidyl-prolyl cis-trans isomerase B	4.0±1.3	yes
NGO0652	thioredoxin I	3.5±1.4	no
NGO0865	unknown	7.8±3.7	yes
NGO1055	acyl-coA hydrolase	10.9±5.0	yes
NGO1771	small conductance mechanosensitive channel	0.8±0.3	yes
NGO1900	OPT-family oligopeptide transporter	2.3±0.8	yes
NGO1947	unknown	3.3±1.2	no
NGO1948	DoxX superfamily	5.9±2.1	yes
NGO2065	UDP-3-O-acyl N-acetylglycosamine deacetylase	6.7±2.4	no

a Open Reading Frame, FA1090 genome annotation.

b mean transcript level after 15 minutes of 5 mM hydrogen peroxide treatment relative to untreated bacteria ± standard deviation.

c recovery of kanamycin resistant colonies after transformation with an EZ-Tn*5* insertion allele.

For genetic complementation of the NGO0322::Tn*5* mutation, a 0.7-kb fragment containing the NGO0322 open reading frame and the predicted promoter region was PCR-amplified from FA1090 genomic DNA and ligated to plasmid pSMART LC Km (Lucigen). The insertion of the intact NGO0322 gene and predicted promoter region was confirmed by sequencing. The insert fragment was then subcloned to the Neisserial complementation vector pGCC5 [36]. The NGO0322::Tn*5* mutant strain was transformed with the complementation construct resulting in a strain harboring a copy of NGO0322 at an ectopic genomic locus. The presence of the intact NGO0322 gene at the ectopic locus was confirmed by PCR.

The NGO0322 mutant and genetic complement lacking *opa* genes were constructed by sequential transformation of the FA1090 Opaless derivative with the plasmid constructs described above containing the NGO0322::Tn*5* and NGO0322^+^ alleles, followed by appropriate genotypic analysis. Constitutive expression of the OpaD protein in Opaless strains was accomplished by transformation using genomic DNA isolated from the OpaD^+^_NV_ strain. Transformants containing copies of the *opaD* ^+^_NV_ allele were identified by their opaque colony morphology and confirmed by PCR.

### Quantitative RT-PCR

The hydrogen peroxide-induced transcription of target genes as previously determined by microarray was confirmed in this study by quantitative RT-PCR. Bacteria were grown in liquid culture to mid-exponential phase and diluted 1∶10. Parallel sets of diluted cultures were either treated with 5 mM hydrogen peroxide or left untreated for 15 min at 37°C. Following incubation, cultures were treated with RNA Protect reagent (Qiagen) and the bacteria collected by centrifugation. Total RNA was purified using the RNeasy kit (Qiagen) according to the manufacturer’s instructions. Purified RNA was treated with RQ1 DNase (Promega) to remove contaminating genomic DNA and reverse transcribed using Superscript III polymerase (Invitrogen). cDNA was amplified using the iQ SYBR Green Supermix (BioRad) according to the manufacturer’s instructions with the following cycling parameters; one cycle, 95°C 3 min.; 40 cycles, 95°C 10 sec., 58°C 30 sec. Relative transcript abundance was determined using the comparative C_t_ method of Schmittgen and Livak [37] with the ROS-independent transcript *omp3* serving as the internal control [29]. All primer sets (sequences available upon request) were confirmed to yield a single amplification product prior to quantitation. Results are the mean fold change in transcript abundance ± the standard deviation of triplicate cultures and are representative of three experiments.

### Infection of Primary Neutrophils

Neutrophils were isolated from venous blood donated by healthy volunteers. All donors provided written consent and protocols were approved by the Northwestern University Institutional Review Board. Briefly, leukocytes were separated from other blood cells by sedimentation using 3% dextran 500 and neutrophils were further separated by centrifugation through a Ficoll-Paque Plus (GE Healthcare) gradient [38]. Remaining red blood cells were lysed by hypoosmotic shock and neutrophils were used immediately after purification. Survival of *N. gonorrhoeae* strains was assayed as described previously using freshly isolated neutrophils and logarithmic-phase bacteria [29]. Neutrophils were seeded onto 13 mm coverslips at a density of 1×10^6^ cells per well in RPMI 1640 (Cellgro) in the presence of 10 nM IL-8 (R&D Systems) for 30 min at 37°C. Bacteria were then washed with RPMI and applied to adherent neutrophils at an MOI of one by centrifugation at 400×g for 4 min. At 0, 30, and 60 minutes post-infection coverslips were washed to remove non-adherent bacteria and neutrophils were lysed by treatment with 1% saponin for 10 min. The number of surviving bacteria at each time point was determined by viable counts using standard procedures.

### Microscopy

Levels of intracellular and extracellular gonococci were quantitated by immunofluorescence as described previously [28], [39]. PMNs were prepared as described for survival assays and seeded onto poly-L-lysine treated glass coverslips. Mid-exponential phase bacteria were added to adherent PMNs at an MOI of one and allowed to incubate for 15 min at 37°C. After co-incubation, non-adherent bacteria were washed away and cells were fixed with 4% paraformaldehyde followed by blocking with 10% normal goat serum. Extracellular bacteria were detected using a rabbit anti-gonococcal primary antibody (Meridian Life Science) and a goat anti-rabbit Alexa Fluor 647-conjugated secondary antibody (Invitrogen). Neutrophils were then permeabilized with 0.2% saponin and intracellular bacteria were labeled with the gonococcal antibody in combination with a FITC-conjugated goat anti-rabbit secondary antibody (Jackson Immunoresearch). Microscopic imaging was performed using a Zeiss LSM 510 laser scanning confocal microscope. Quantitation of intracellular and extracellular bacteria was determined by counting all cell-associated bacteria from 30 fields for each infection condition. The number of neutrophils counted ranged from 81–114.

### Quantitation of the Neutrophil Oxidative Burst

The generation of a neutrophil oxidative burst was measured by luminol-dependent chemiluminescence. Suspensions of 1×10^6^ PMNs in RPMI lacking phenol red were seeded into 96-well plates containing 100 µM luminol reagent (Sigma-Aldrich). Mid-exponential phase bacteria were then added at an MOI of ∼10 and luminescence was monitored on a SpectraMax M5 plate reader (Molecular Devices) every five minutes for one hour.

### Detection of Opa Protein Variation

Eight of the 11 FA1090 Opa variants are antigenically distinct and therefore Opa variants in this study were detected by immunoblot. PBS suspensions of bacteria from agar plate growth were normalized according to optical density prior to lysis at 100°C for 5 minutes in solubilization buffer. Bacterial lysates were separated on 15% SDS-PAGE gels followed by transfer to Immobilon PVDF membranes (Millipore) using standard techniques [40], [41]. For general detection of Opa proteins, lysates were subjected to western blotting using a cocktail of previously described monoclonal antibodies capable of recognizing the OpaA, B, C, D, E, F, and K variants [42]–[44] or the pan-Opa monoclonal antibody 4B12 [45]. Detection of specific Opa variants was accomplished by western blot using individual polyclonal antibodies raised against peptide fragments of OpaA, B/D, C, F, and I [46] or the OpaE/K monoclonal antibody described above [22], [44].

## Results and Discussion

### Construction of Mutations in Peroxide-inducible Genes

The microarray analysis that defined the hydrogen peroxide regulon of *N. gonorrhoeae* led to the identification of genes that when mutated resulted in increased sensitivity to hydrogen peroxide and/or neutrophil-mediated killing, but the neutrophil killing was by nonoxidative means [26], [29]. In this study, we sought to identify additional hydrogen peroxide-induced genes that function in bacterial resistance to neutrophil-mediated killing. Several genes were selected for analysis based on their predicted function and degree of transcriptional activation following hydrogen peroxide treatment. Quantitative PCR was performed to confirm the previously reported microarray results for each of the candidate genes. The majority were confirmed to have elevated transcript levels following exposure of bacteria to 5 mM hydrogen peroxide for 15 minutes, with the exception of NGO1771, the expression of which was not significantly altered by treatment (Table 1).

Each of the genes listed in Table 1 was targeted for mutation in order to test for involvement in defense against neutrophil-mediated killing. A cloned copy of each gene was disrupted by insertion of a Tn*5*-based transposon conferring kanamycin resistance. The mutant alleles were subsequently transformed into *N. gonorrhoeae* and transformants were selected by resistance to kanamycin. Transformation of gonococci with constructs containing insertions in open reading frames NGO0108, NGO0652, NGO1847, and NGO2065 failed to yield kanamycin resistant colonies. Based on the lack of transformants, these genes have the potential to encode essential products, and they were not pursued further. Strains containing mutations in the remaining eight genes in Table 1 were tested for sensitivity to hydrogen peroxide but none were found to be significantly different than the parent strain (data not shown). Although the function of this subset of hydrogen peroxide-induced genes remains to be determined, this result is consistent with the hypothesis that their physiologic role is not in protection from oxidative stress.

### Misidentification of NGO0322 as a Novel Virulence Factor

**Figure 1:**
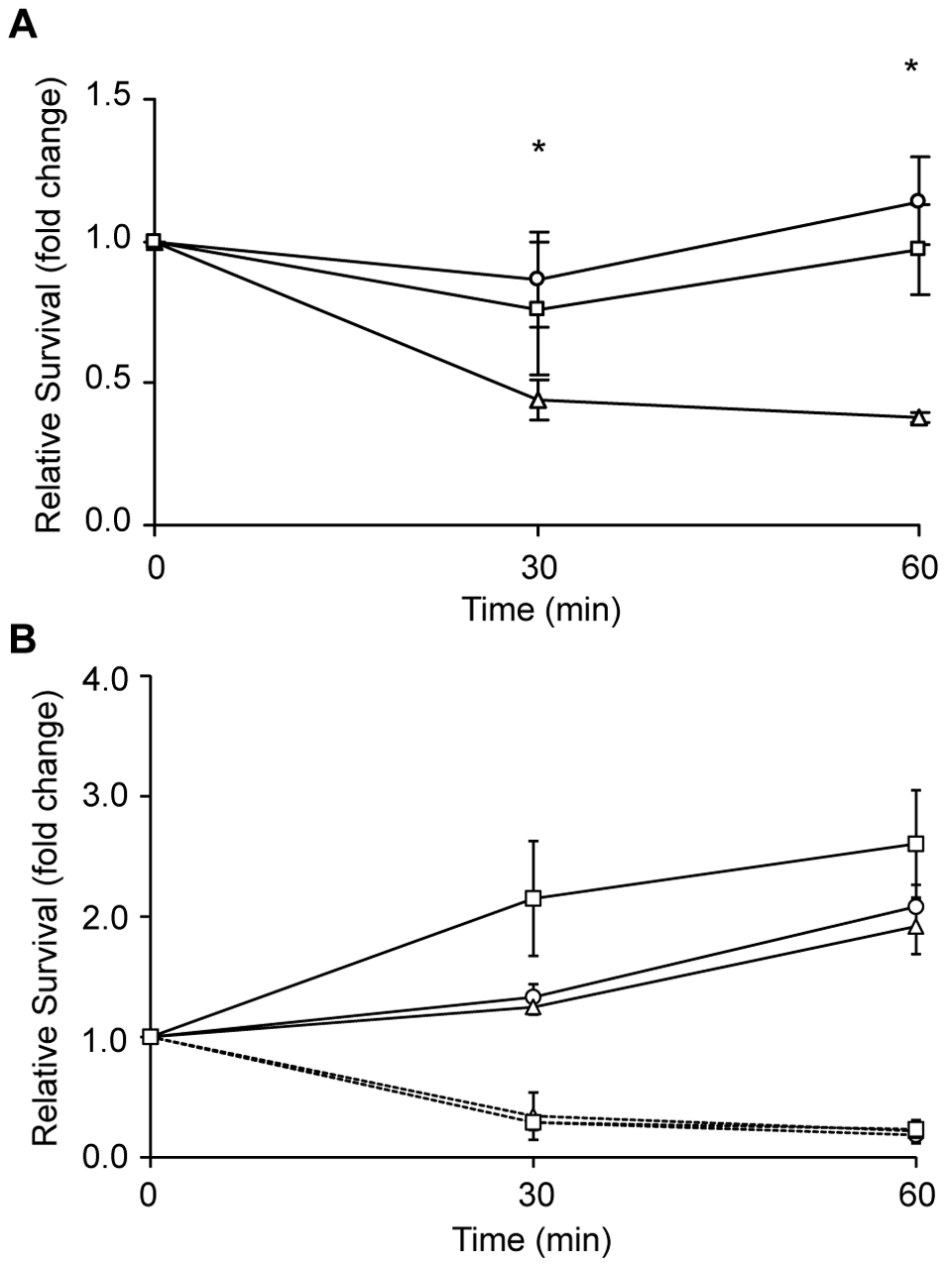
Neutrophil sensitivity of *N. gonorrhoeae* strains harboring NGO0322 mutations. Primary human neutrophils were isolated from donor blood and infected with *N. gonorrhoeae* strains at an MOI of one. Neutrophil-mediated killing is reported as the number of bacterial CFU recovered at each time point relative to time zero. **A**. Neutrophil-mediated killing of *N. gonorrhoeae* Opa phase-variable strains: circles, FA1090 NV parent; triangles, NGO0322::Tn*5* mutant; squares, NGO0322::Tn*5*, NGO0322^+^ genetic complement. Asterisks indicate a statistically significant difference in the survival of NGO0322 mutant bacteria compared to that of the parent strain as determined by Student’s t-test, p<0.005. **B**. Neutrophil-mediated killing of Opaless and OpaD^+^_NV_ NGO0322 mutant derivatives. Relevant NGO0322 genotype of *N. gonorrhoeae* strains: circles, NGO0322^+^ (parent); triangles, NGO0322::Tn*5*; squares, NGO0322::Tn*5*, NGO0322^+^. Solid lines indicate the results from strains that were derived from the Opaless strain lacking all Opa proteins. Dashed lines indicate overlapping results from all three Opaless derivatives that constitutively produce the OpaD protein (OpaD^+^_NV_). The results are representative of at least three experiments and error bars indicate the standard deviations from the mean of triplicate determinations.

The notion that at least a subset of hydrogen peroxide-induced genes are not involved in resisting oxidative stress is consistent with the observation that neutrophil-mediated killing of *N. gonorrhoeae* is independent of the neutrophil-generated ROS [24]–[26], implying that these gene products may be involved in resisting alternative killing pathways. To test the contribution of the genes in Table 1 to neutrophil resistance, viable mutants were used to infect primary neutrophils isolated from human volunteers. The survival of each strain was compared to that of the FA1090 NV parent strain during a one hour infection. For strains harboring mutations in the NGO0114, NGO0376, NGO0865, NOG1055, NGO1771, NGO1900, and NGO1948 open reading frames, no consistent difference in neutrophil sensitivity was observed in comparison to the parent strain (data not shown). The viability of a single strain, NGO0322::Tn*5*, was consistently attenuated in the presence of neutrophils (Fig. 1A). At 30 minutes post infection there were significantly fewer viable NGO0322::Tn*5* bacteria relative to time zero compared to the parent strain. The survival defect of NGO0322::Tn*5* was maintained throughout the course of infection whereas the parent strain was able to overcome neutrophil-mediated killing and replicate by 60 minutes. An isogenic derivative of the NGO0322::Tn*5* mutant containing a copy of NGO0322 at an ectopic chromosomal location exhibited a phenotype in which survival levels were largely restored to that of the wild-type strain. Although ultimately proven incorrect, these data provided convincing evidence that NGO0322 functioned in providing resistance to neutrophil-mediated killing.

**Figure 2:**
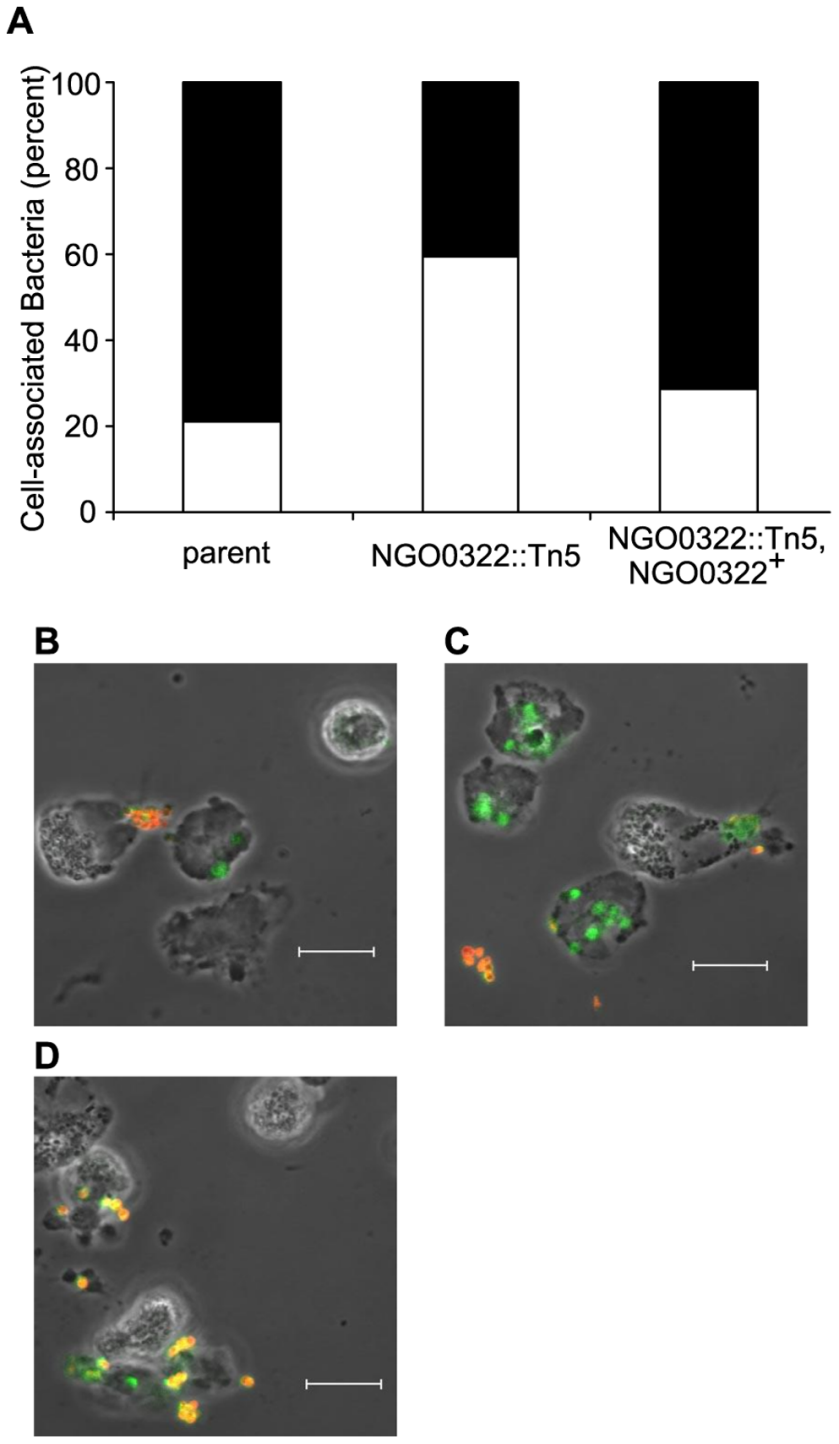
Enhanced neutrophil internalization of Opa-variable NGO0322 mutant bacteria. The cellular localization of *N. gonorrhoeae* strains was determined by differential staining of intracellular and extracellular bacteria after infection of human neutrophils. Bacteria were detected using an anti-gonococcal primary antibody before and after neutrophil permeabilization. Extracellular bacteria were labeled with a secondary antibody conjugated to Alexa Fluor-647 and appear yellow or orange, while intracellular bacteria were labeled with a FITC-conjugated secondary antibody and appear green. **A**. The ratios of cell associated intracellular (white bars) and extracellular bacteria (black bars) are shown from 30 randomly selected fields for the given *N. gonorrhoeae* strains. The results are representative of three independent experiments. Representative images for strains FA1090 NV (**B**), NGO0322::Tn*5* (**C**), and NGO0322::Tn*5*, NGO0322^+^ (**D**) are also shown.

Additional evidence for the importance of NGO0322 during neutrophil infection was obtained by determining the localization of cell-associated bacteria. Adherent IL-8 treated primary neutrophils were infected with the *N. gonorrhoeae* NGO0322::Tn*5* mutant and control strains and the intracellular or extracellular location of bacteria was differentiated by immunofluorescence. Following a 15 minute incubation, all three strains of bacteria could be found both intracellularly and extracellularly associated with neutrophils. However, a higher proportion of NGO0322::Tn*5* mutant bacteria were intracellular compared to the parent strain or the complemented mutant (Fig. 2). These results suggested that either NGO0322::Tn*5* mutant bacteria were internalized more readily than the NGO0322^+^ control strains or that NGO0322 mutants were more sensitive to extracellular neutrophil killing mechanisms. It was also possible that these results were due to the presence or absence of Opa proteins that mediate internalization by neutrophils [23].

**Figure 3:**
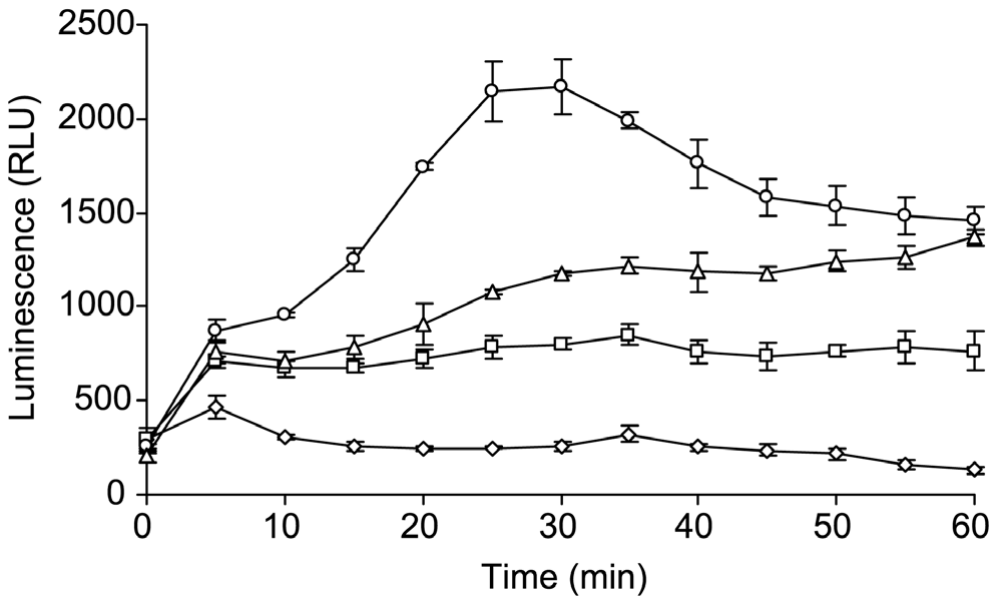
Enhanced ROS production by Opa-variable NGO0322 mutant bacteria. Primary human neutrophils were infected at an MOI of 10 in the presence of luminol reagent and the production of ROS by neutrophils was monitored by chemiluminescence. Relative light units (RLU) are reported as the means of triplicate determinations ± the standard deviation and are representative of the results from three experiments. Assay conditions: diamonds, neutrophils alone; squares, FA1090 NV parent; circles, NGO0322::Tn*5* mutant; triangles, NGO0322::Tn*5*, NGO0322^+^ genetic complement.

Finally, the higher proportion of intracellular NGO0322 mutant bacteria relative to the NGO0322^+^ strains suggested that the NGO0322 protein product was manipulating the neutrophil response to bacterial infection. To test whether this altered response resulted in modulation of the neutrophil oxidative burst, cells in suspension were infected with NGO0322 mutant or control bacteria at an MOI of 10 and the generation of oxidants was monitored by luminol activity. Uninfected neutrophils exhibited a low level of baseline activity whereas neutrophils that were infected with the parental *N. gonorrhoeae* strain showed a consistently low, but slightly elevated, level of respiratory activity throughout the 60 minute infection (Fig. 3). In contrast, infection of neutrophils with NGO0322 mutant bacteria resulted in an increase in luminol activity of ∼2.7-fold compared to that of the wild-type strain at 25 minutes post-infection. Complementation of the NGO0322 mutation resulted in a partial restoration of the parental phenotype with luminol activity being significantly lower than that resulting from infection with mutant bacteria throughout much of the time course. Regardless of the genetic change in the NGO0322::Tn*5* isolate that resulted in enhanced neutrophil ROS production, these observations were consistent with the increased internalization of this strain (Fig. 2), yet do not account for the increased susceptibility to neutrophil-mediated killing as survival was unaffected by pretreatment of neutrophils with the NADPH oxidase inhibitor diphenyleneiodonium (data not shown).

### Opa Protein Profiles of Isogenic NGO0322 Strains

**Figure 4:**
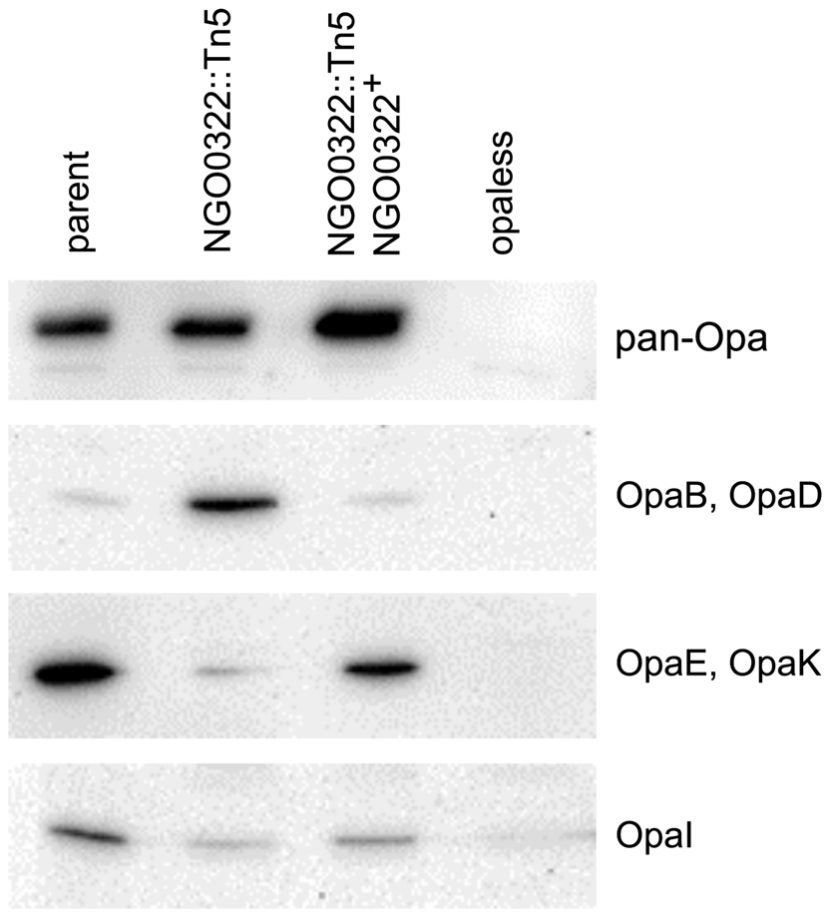
Differential Opa protein production by NGO0322 mutant and control strains. Lysates from bacterial suspensions normalized by optical density were separated by SDS-PAGE and tested for the presence of Opa proteins by western blot. Variant-independent detection of Opa proteins was accomplished with the pan-Opa monoclonal antibody 4B12. Primary antibodies used in the detection of specific Opa proteins were immunoreactive to variants OpaB and OpaD, OpaE and OpaK, or OpaI. The Opaless strain harbors mutations in all 11 *opa* alleles and exhibited no reactivity to any of the antibodies tested, all other strains shown in this figure were capable of Opa phase variation.

*N. gonorrhoeae* strains expressing surface exposed Opa proteins stimulate an oxidative response from primary neutrophils while gonococci lacking Opa do not induce a respiratory burst but rather suppress ROS production in the presence of exogenous stimuli [28]. Similarly, Opa proteins are known to sensitize gonococci to neutrophil-mediated killing in the *ex vivo* assay system [26]. Even though genetic complementation of the NGO0322 gene resulted in at least partial restoration of the parental phenotype in terms of neutrophil sensitivity, internalization, and ROS production, it remained possible that these phenotypes were due to differential Opa production. When tested for reactivity to the pan-Opa monoclonal antibody 4B12, all three relevant strains were strongly positive by western blot (Fig. 4). In addition, Opa production was determined by using individual antibodies with specificity for the immunologically distinct members of the FA1090 Opa repertoire. Antibodies with reactivity toward OpaA, OpaC, and OpaF yielded no signal when lysates of the parent strain, NGO0322 mutant, or the genetically complemented strain were tested (data not shown). However, there were distinct differences in the OpaI, OpaB/D, and OpaE/K profiles of these strains, with the NGO0322 mutant and NGO0322^+^ strains having opposing phenotypes with regard to OpaB/D and OpaE/K antibody reactivity and all strains showing low but heterogeneous levels of OpaI (Figure 4). Given the inconsistency in the Opa phenotype of these strains, it was possible that the differential neutrophil sensitivity observed between NGO0322 mutant and NGO0322^+^ strains was independent of NGO0322 function.

### NGO0322 does not Contribute to Neutrophil Sensitivity

To investigate the contribution of NGO0322 to gonococcal PMN sensitivity it was necessary to eliminate the inherent variability provided by Opa phase variation. Isogenic NGO0322 mutant stains were created in a previously constructed FA1090 strain in which each of the 11 *opa* genes had been disrupted, resulting in the complete absence of Opa proteins (Opaless) [19]. The Opaless NGO0322 mutant exhibited no loss of viability during PMN infection, similar to the parent strain and the Opaless NGO0322 genetic complement (Figure 1B, solid lines). These results strongly indicate that NGO0322 does not contribute to survival of gonococci during PMN infection. It is clear from the results in Figure 1A and Figure 4 that the general presence or absence of Opa proteins does not account for the strain-to-strain variation in PMN sensitivity observed with the Opa^+^ NGO0322 mutant strain. Rather, differential expression of Opa proteins (B, D, E, K, or I) is likely to account for the PMN-related phenotypes of the Opa phase variable strains tested. Therefore, assessment of Opa protein production between strains using a pan-Opa antibody is insufficient to assure that variable Opa production does not influence interactions with host cells. Although it remains to be determined which of these 5 proteins is responsible for the PMN sensitivity phenotype of the NGO0322 mutant, this result is likely due to altered neutrophil response to the Opa variants produced by each strain. Of note, OpaD has previously been demonstrated to bind CEACAM receptors and stimulate high levels of ROS production, beyond that of other FA1090 Opa variants [19], [47], suggesting that OpaD expression results in a more robust neutrophil response. The three Opa-binding receptors that have been identified on human neutrophils are CEACAM1, CEACAM3, and CEACAM6, with CEACAM3 expression being unique to this cell type [48]. Opa binding to CEACAM3 in particular has been demonstrated to stimulate neutrophil ROS production and degranulation, while all three relevant CEACAMs are capable of mediating bacteria internalization [23].

It remained formally possible that the NGO0322::Tn*5* phenotype required the presence of Opa proteins, and thus no neutrophil sensitivity would be observed in the absence of Opa proteins. To address this possibility, each of the Opaless strains was transformed with an allele containing the *opaD* gene with synonymous mutations in the CTCTT phase-variable repeat region (OpaD^+^_NV_) [19], such that each of the strains was constitutively OpaD^+^. Figure 1B (dashed lines) shows that the NGO0322::Tn*5*, OpaD^+^_NV_ strain had indistinguishable neutrophil sensitivity from the NGO0322^+^, OpaD^+^_NV_ control strains, eliminating the possibility that Opa production was necessary for the NGO0322 phenotype. It should also be noted that the OpaD^+^_NV_ strains were dramatically more sensitive to PMN-mediated killing than the Opaless strains, reinforcing the conclusion that expression of Opa proteins, and OpaD in particular, significantly alters the ability of *N. gonorrhoeae* to withstand neutrophil-mediated killing. In contrast, robust expression of the OpaE or OpaK variants by the FA1090 NV parent strain (Fig. 4) was not sufficient to cause dramatic loss of viability or high frequency internalization in the presence of neutrophils (Figs. 1A and 2). These results support the conclusions from previous works that indicate different Opa variants do not contribute equally to host cell responses [19], [23], [47], [49].

### Conclusions

The objective of this work was to identify novel *N. gonorrhoeae* genes required for gonococcal resistance to neutrophil-mediated killing. This was based on the hypothesis that ROS produced by neutrophils in response to gonococcal infection stimulate transcription of *N. gonorrhoeae* genes that are required to combat the antibacterial arsenal of innate immune cells. From a panel of 12 hydrogen peroxide-inducible candidate genes we identified NGO0322 as meeting the criteria of a gonococcal virulence factor during neutrophil infection. Despite meticulous consideration for the inherent stochastic variability of gonococcal surface constituents, such as Opa proteins and type IV pili, as well as the implementation of standard genetic controls in all experiments, the identification of NGO0322 as a novel virulence factor was in fact due to differential Opa production by the strains used in this study. This was conclusively demonstrated by assessing the contribution of NGO0322 to neutrophil resistance in strains that were incapable of Opa production and strains that constitutively express only the OpaD protein. The other seven viable mutants generated in this study did not exhibit differential sensitivity to neutrophil-mediated killing; however, because these strains were capable of Opa phase variation the involvement of these genes in neutrophil interactions cannot be eliminated.

Although ultimately failing to confirm the initial hypothesis, the results from this work serve to highlight the importance of gonococcal Opa proteins in neutrophil interactions and the necessity for ensuring a consistent Opa phenotype, either genetically or through tracking expression of individual Opa proteins, when investigating interactions of *N. gonorrhoeae* with host cells. Lipooligosaccharide, though not investigated here, represents another biologically important surface molecule that is subject to phase variation in this and other organisms [9], [10], [50], [51] and has the potential to alter interactions between pathogenic bacteria and host cells in model systems. The extensive phase variable repertoire of this and other bacterial species represent a common means by which bacteria generate spontaneous phenotypic diversity that can also present practical difficulties for studying these organisms in the laboratory.
